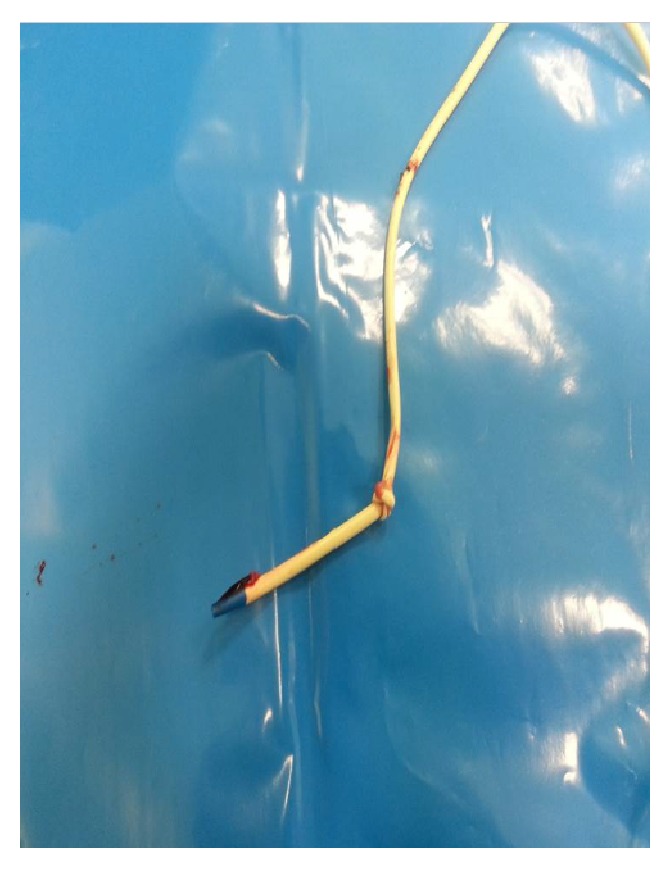# Jugular Venous Catheterization: A Case of Knotting

**DOI:** 10.1155/2015/810346

**Published:** 2015-11-08

**Authors:** E. Erkılıç, M. Aksoy, T. Gümüş, S. Sarı, E. Kesimci

**Affiliations:** ^1^Anesthesiology and Reanimation Department, Ataturk Training and Research Hospital, Ankara, Turkey; ^2^Faculty of Medicine, University of Yıldırım Beyazıt, Ankara, Turkey

## Abstract

A 79-year-old woman, diagnosed for cancer of the ovary, had a central catheter that was placed with difficulty through the right internal jugular vein intraoperatively. After oophorectomy, it was realized that the catheter was knotted. Thus, the central venous catheter was removed successfully using a traction technique in the operating room. Central venous catheter use may result in various complications, although it has been used as an invasive method for hemodynamic monitoring and fluid and drug infusion. Here, we present catheter knotting in a case with solutions for this complication, under literature review.

## 1. Introduction

Central venous catheterization is an invasive intervention used for drug administration and hemodynamic monitoring both in operating rooms and in intensive care units. The frequency of complications is between 5 and 29% [1]. Case reports regarding catheter malposition have already been published [2]. We have encountered cases of knotted guide wire in the literature review [3].

We herein aimed to present our approach to a case of knotting of a central venous catheter on itself, which was not realized until its removal.

## 2. Case

**Figure 1:**
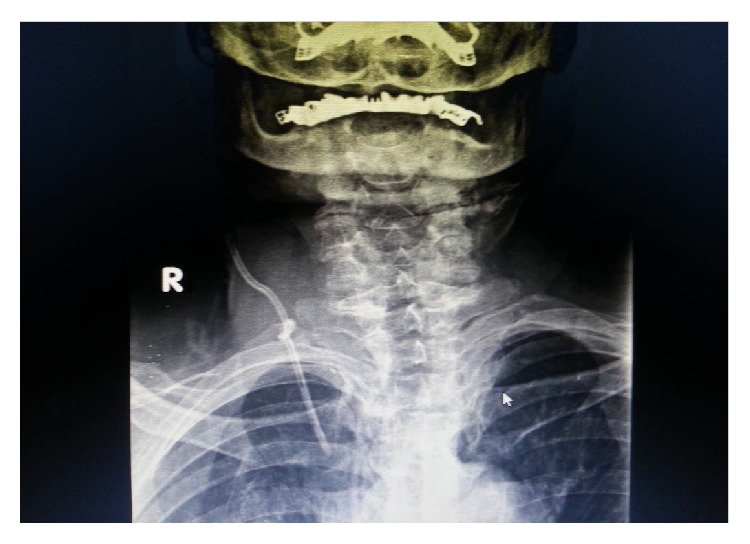


**Figure 2:**
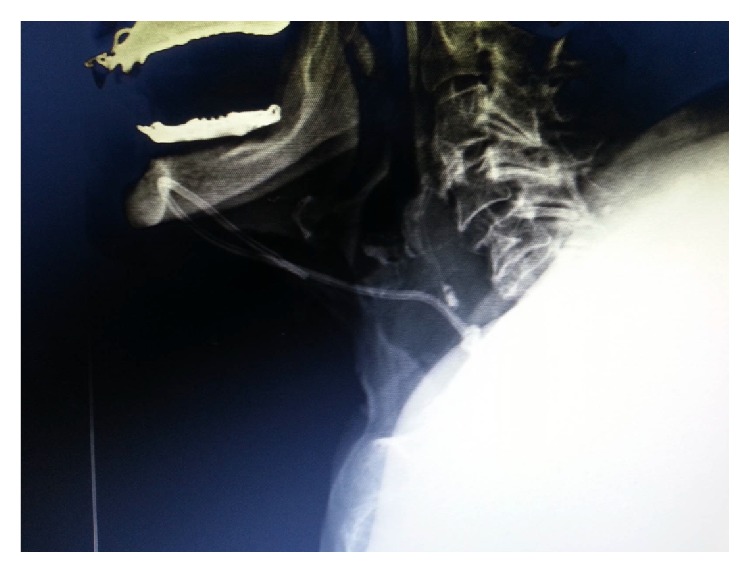


The preoperative evaluation of a 79-year-old female patient who was 83 kg with a short neck was nonspecific, other than hypertension. She was scheduled for oophorectomy. The patient was taken to the operating room without premedication and standard monitoring was applied (ECG, SpO_2_, and noninvasive blood pressure). Intravenous access was achieved by inserting a 20 G intracatheter. After preoxygenation, anesthetic induction was performed using lidocaine and thiopental sodium and tracheal intubation was achieved by rocuronium bromide using an appropriate size endotracheal tube. In addition to 50% O_2_/air, 0.125–0.25 *μ*g·kg^−1^ remifentanil infusion and 0.5–1% sevoflurane were used in the maintenance of anesthesia. The right radial artery was used for invasive intra-arterial cannulation and monitoring of arterial blood pressure following anesthesia induction. Central venous catheterization was successful on the fourth attempt through the right internal jugular vein using an appropriate size central catheter (arrow multilumen central venous catheter: 7 Fr). The resident could not puncture the vein at first. Then in the second trial he had difficulty in advancement of the guide wire following a successful puncture. Then the consultant anesthetist tried. In his first attempt, the guide wire could not be advanced. Lastly he put the catheter into the internal jugular vein. We had the central catheter for a possible blood transfusion and central venous pressure monitoring intraoperatively. Blood flow was observed through all lines of the catheter without any difficulty and all lines were irrigated. Subsequently, the surgical procedure was allowed to proceed without having chest X-ray. On the other hand, we did not use ultrasound imaging for either catheterization or correction of its place. The hemodynamics of the patient deteriorated because of the hemorrhage from the surgical site and arterial blood pressure fell to 80/50 mmHg intraoperatively. The heart rate was stable, but it was decided that the lost blood volume was to be replaced. Thus the hemoglobin control resulted in 9 g·dL^−1^. Since the duration of the operation was extensive and acidosis developed, the patient was transferred to the intensive care unit (ICU) to be followed up. In ICU, the central venous catheter was used also for normal intravenous crystalloid and drug, antibiotic therapy. There was no problem in the speed of infusions. There were no signs of extravasation. Thus, the intensivist was not suspicious of a problem in the catheter. He did not check it with chest X-ray. The patient remained on mechanical ventilator support for one night and was extubated later. Since the patient's hemodynamics were stable and she was conscious, after 24 hours of intensive care treatment, she was transferred to the service. The patient had poor and fragile peripheral veins. Thus, we left the catheter in its place while discharging her from ICU. During her follow-up in the Gynecology Service, the catheter was used as an intravenous route for crystalloid infusion and antibiotherapy. Her central venous catheter was decided to be removed on the postoperative sixth day since oral intake was adequate and the patient was mobile. While removing the catheter, resistance was noted and the catheter could not be withdrawn; therefore it was left in place without application of any force. Posteroanterior and lateral chest X-rays were obtained and the catheter was observed to be knotted in a location close to the subcutaneous tissue (Figures 1, 2, and 3). The patient was transferred to the operating room and the catheter was withdrawn by the standby of cardiovascular surgery team without any difficulty and a need for surgery.

## 3. Discussion

Central venous catheters have been used extensively for hemodynamic monitoring, drug administration, hemodialysis and/or hemofiltration, and cardiac pacing. Large bore central venous catheters are also advantageous in rapidly infusing resuscitation fluids [4]. Complications associated with the central venous catheters carry risks for the patients and the treatment of those complications is expensive [5]. Complications are observed in 15% of the patients. Among these, mechanical, infectious, and thrombotic complications are reported in 5–19%, 5–26%, and 2–26% of the cases, respectively [6]. The Seldinger technique has widely been used for central venous catheterization [7, 8].

Complications related to guide wire use include kinking, knotting, looping, breakage, and fracture of the catheter [9–14]. Broken or fractured guide wire fragments might result in severe consequences, such as embolization or even cardiac arrest [15, 16]. Guide wire fragments can be removed with a snare loop catheter that is inserted through the femoral vein [17]. Another rare complication observed is intravascular knotting of the catheter. In this patient we had 4 attempts to succeed in the catheterization. As we are in a teaching hospital, the residents are allowed to catheterize the patients under the observation of their consultants. In the literature, there are reports about the number of percutaneous punctures per attempt significantly associated with complication rates [18]. One of the largest studies described that attempts requiring more than 2 punctures had an incidence of 43% failure rate and a mechanical complication rate of 24% [19]. Eisen et al. said that the residents and interns attempted 80% of the CVCs, and only 3% of these attempts were under the control of the consultants. As the author suggested, the selection of the patients for proper placement of the catheters is very important [20]. Probably, the patient in our case was not appropriate for a resident's trial due to anatomical disadvantages. However the standby of the consultant encouraged him to try twice. Besides, we were not suspicious of any complications. Knotting of an intravascular catheter was reported for the first time in 1954 by Johansson et al. [21]. More than 2/3 of the reported cases of catheter knotting occur in pulmonary artery catheters, especially. This is because the pulmonary artery catheters are thinner, more flexible, and longer and there is no guide wire present that is used to advance the catheter [22]. For checking tip position during the insertion, intracavity ECG or ultrasound might be used besides X-ray [23]. Although intracavity ECG is accurate, well tolerated, and cost-effective, there may be problems with equipment as we have in our hospital. It is not meaningful when the P wave is not detectable, for example, in atrial arrhythmias [24]. McGee and Gould suggested the use of ultrasound guidance as a method of reducing the risk of complications during central venous catheterization [1].

In this case, blood was withdrawn easily and the infusions continued undisturbed through the catheter, so we did not suspect of a presence of any complication. In spite of the adequate experience of the doctor who performed the cannulation, the reason for the difficulty in the insertion was assumed to be due to difficult anatomy. We did not check its place by X-ray intraoperatively, because we allowed the surgeons to start surgery as quick as possible. It is likely that the knot was floppy during insertion and it permitted the blood draw and intravenous infusions. However, it was postulated that the knot of the catheter was tightened during the first attempt of withdrawal of the catheter. When the type of occurrence of the knot was analyzed, the J end of the guide wire was thought to be hooked and it caused the catheter to make a loop. Therefore, the knotted catheter was easily observed by chest X-ray (Figure 1). Some authors report that chest X-ray alone is not sensitive and specific enough to decide whether the catheter tip is placed in proper position [25]. Additionally, some authors recommend monitoring patients after catheter insertion and perform delayed chest X-ray in the presence of any complication [26]. Most cases of knotting can be resolved using simple maneuvers. However, some special techniques have also been developed in cases in which the catheter removal is difficult. One of the preferred techniques among those is to attempt to undo the knot by sending a guide wire through the catheter [27]. However, to undo the knot by this technique frequently fails when the knot is far from the skin site or when the knot is so tight that it renders the passage of a guide wire impossible. The removal of the catheter from the insertion site by applying traction is also a method described earlier [28]. However, the possibility of injury in the internal jugular vein or the subclavian vein should be considered in this case. Karahan et al. identified three cases of knotting in their series of 2310 cases of pulmonary artery catheterization. Two of the catheters sited above were removed by reducing the knot through a percutaneous transvenous technique; however, an open surgical technique was required in one case [29, 30]. Reports of removal of the central catheters without any problem by performing a venotomy under local anesthesia have been published [29]. Georghiou et al. encountered resistance during the withdrawal of a Swan-Ganz thermodilution catheter [30]. Before the trial of any traction method, they directly performed sternotomy and surgical approach and noticed that the catheter was in the superior vena cava.

Although development of a knot is a rare complication, preventing the occurrence of this complication is as important as solving the problem itself. Therefore, both clinicians and anesthesiologists who perform central venous or pulmonary catheterizations should keep their knowledge updated about prevention of complications and treatment options, in order to prevent the morbidity and mortality. Routine checks should not be omitted in such invasive procedures. We suggest ultrasound guided catheterization in patients with difficult anatomy, as well as radiological examination during the early postoperative period.

## Figures and Tables

**Figure 3:**